# The Effects of Lightning, Fire, Smoke and Steam upon Animals

**Published:** 1886-07

**Authors:** Thomas Walley

**Affiliations:** Principal Royal (Dick) Veterinary College, Edinburgh


### ART. XIV.—THE EFFECTS OF LIGHTNING, FIRE, SMOKE AND STEAM UPON ANIMALS.

BY THOMAS WALLEY, M.R.C.V.S., PRINCIPAL ROYAL (DICK) VETERINARY COLLEGE, EDINBURGH.

There is, probably, no subject of more varied interest to the veterinary surgeon than the above; there is certainly no veterinary medical subject upon which so little attention has been bestowed. It is not my intention to enter, in this article, upon the discussion of theoretical or new points, but rather to deal with the practical aspect of the question, and to give to the profession the lessons I have learned by practical experience.

### LIGHTNING.

THE EFFECTS OF LIGHTNING upon the animal frame are practically those of electricity, and are dependent upon the intensity of the electric current. These effects may be divided into *immediate* and *remote*. If the intensity of the electric current is sufficiently great, DEATH BY SHOCK is the immediate result. If it is of less intensity, death also results; but much less quickly, and from GENERAL OR CARDIAC ASTHENIA, and in consequence of the effects of the electricity upon the great nerve centers. In the former case; there is sudden and total abolition of nerve function; in the latter, there is inability to rally from the primary effects of the nervous shock, and in addition, grave changes are produced in the vital properties of the blood.

While the effects above mentioned are, ordinarily, those of exposure to the influence of lightning, it is not uncommon to find that animals pass through the ordeal unscathed and unscarred; or, at the most, suffer from TEMPORARY BLINDNESS from the effects of the intense glare upon the retinal expansion of the optic nerve. The AMAUROSIS so produced occasionally becomes permanent, owing to the continued paralysis of the nerve.

*The remote effects upon the nervous system and consequently upon the muscular* are PARESIS and MOTOR PARALYSIS; the former may affect particular muscles or sets of muscles, as for example, one of the labial or facial; while in the latter case NEMIPLIGIA, or PARAPLEGIA may result.

In some instances, grave cutanio muscular lesions are produced; the skin and subjacent structures either being destroyed chemically, or actually lacerated; resulting, if the animal survives, in extensive *sloughing.* This being so great in some cases as to produce death by secondary exhaustion.

The post-mortem evidences of lightning stroke are by no means constant, nor are they in all cases satisfactory, and it often happens that the veterinary surgeon, when called upon by insurance companies to make post-mortem examinations of animals supposed to have died from the effects of lightning, has to trust mainly to *negative* and *collateral evidence* to enable him to arrive at a just and correct decision. Negatively, there will be an entire absence of any lesion sufficient to account for death, and, at the same time, proof may be forthcoming that the animal or animals had been noticed a short time prior to death in a state of perfect health, and that, in the interim, a thunder storm had passed over the district.

Collateral evidence is, as a rule, more satisfactory than negative,—it is to be found in the surroundings of the victim or victims. Thus, if the animal has been sheltering behind a hedge, the bushes comprising the latter will bear evidence of the lightning flash in the form of scorched leaves and branches, or, if the hedge bottom be very dry, it may afford evidence of recent ignition. The banks, too, are often ploughed up and torn into furrows by the electric fluid, while leaves, torn from their branches, and twigs of trees or bushes will be found scattered about the surrounding turf or soil. When shelter has been sought under a tree, indubitable evidence of the action of the electric fluid—in the shape of splintered limbs and branches, torn-up bark, and even roots—is afforded; while the victim (and this holds good no matter how many animals there may have grouped together) is found with its head lying in close proximity to the tree trunk.

In the case of a shod horse—even if standing in an open field when struck—one or more shoes may be found torn from the feet, and the iron itself fused or distinctly magnetized. The carcasses of animals have also been observed to be magnetized by the action of the electric current.

*The more positive signs of death by lightning* are to be found on the exterior of the body and in the condition of the blood, the muscles, and some of the internal organs. On the skin, the course of the electric fluid can often be traced without the slightest difficulty; the hair, in hairy animals, being scorched in zig-zag lines, commencing often at the shoulder or neck and running down to the foot (as if attracted, in the shod-horse, by the metal of the shoe) and into the earth.

In the sheep, the external evidence is much less prominent than it is in the ox or the horse. Wool is apparently a non-conductor of electricity, and I am led from personal observation to believe that if the face of the sheep had, like its body, been protected by wool but few cases of death by lightning would occur among the ovine species. In every instance that has come under my observation, I have found unmistakable traces of the point of impact on the skin of the face, of the electric fluid having taken its course thence, not on the surface of the wool, but along the skin itself—the roots of the wool being discolored (usually brown or yellow) and shrivelled. In some cases, the wool along the track of the fluid may be, as it were, seperated.

The arborescent markings and the mapping out of the course of the nerves and blood vessels—caused by the reduction of the hœmoglobin of the blood—so frequently seen on the body of man are much less pronounced on the skin of animals; nevertheless, such markings can be detected by dissection in the skin itself, and in the sub-cutaneous tissues.

True *rigor mortis* does not, in the vast majority of instances, take place in death by lightning, but the carcass usually retains the position assumed by the animal on its falling to the earth, and, as before remarked, when animals have been sheltering under a tree, the bodies are found grouped in a circle round its trunk, with the heads towards the center of the circle. After the lapse of a few hours the carcass becomes stiff from the liberation of gases, and the body and legs quite rigid; the latter are extended in a straight line. The body is tympanitic, blood or bloody foam oozes from the nostrils, the rectal mucous membrane becomes everted, and there may even be oozing of blood from the anus.

The blood, it is generally assumed, does not coagulate, and its hernate-globului becomes dissolved. Coagulation of the blood does, however, take place in some instances, and cardiac clotting is occasionally seen. Pulmonary congestion pronounced in some cases, but neither cerebral nor spinal lesions are, necessarily, to be detected.

*Vagaries in the continuity of lightning-stroke* are sometimes remarkable, but these vagaries can generally be explained by natural influences. Thus, indirect transmission may may be accounted for by a break in the line of contact of animals with the conducting medium. A good example of this is recorded in the case of a number of horses struck by lightning in the cavalry stables of Krasnoje-Selo, in 1883. In this instance, sixteen horses were killed outright, their companions suffering from temporary loss of muscular power only, and it was observed that, in the case of the dead animals, the electric fluid had struck the head or neck and passed downward to the fore feet. The hay racks were bound with iron, and their contents were burned, and it was assumed that the victims (picked out here and there) had, at the moment the electric fluid struck the iron, been standing with the head in contact with, or in close proximity to the iron.

In 1886, a case occurred at Camilla, New York, in which five horses, standing with their heads over a wire fence, were instantaneously killed. While an instance of remarkable escape from death is recorded in the *Lancet*, of January 11, 1879, in the case of a man from whose body the clothes were stripped; his watch, the buttons of his clothes and other metals were fused; and yet he, himself, suffered only from temporary injury.

### FIRE.

In speaking of DEATH BY FIRE it must be borne in mind that there are other factors in operation to produce death, besides actual burning. Undoubtedly, actual cauterization is the most potent agent in the production of death and of severe injuries in animals exposed to the influence of fire, but there are usually other agencies in operation tending to the same end. Thus a fire may take place in a large building, within the walls of which horses, cattle and other animals may be housed, and after the extinction of the fire a number of them may be found dead, and yet in no case will traces of actual burning be discovered on their bodies. This is a common experience, and to those unversed in such matters, it seems, at first sight, quite inexplicable: a little thought and investigation will soon supply the explanation.

I have seen instances of fire breaking out in a stable, where the flame has swept along the edge of the bedding of each individual animal, and in which scarcely a mark was to be discovered in either stall posts or horses, but a number of the horses have been severely and even irrevocable injured.

I have seen one instance where the flame ran along a continuous hay-rack, and in which the hay was consumed and the rack charred, yet not one single horse was deprived primarily of life, but some were rendered either temporarily or permanently useless.

Again, I have seen a part of a stable consumed by fire, but out of five or six horses deprived of life, no trace of fire could be found on the carcass of any individual animal.

The explanation of all this is, that in these cases in which animals are not actually burned to death many of them die from SHOCK, or, it may be better expressed perhaps, from FRIGHT. I am quite convinced that in some of these instances SUDDEN CARDIAC PARALYSIS takes place.

In the vast majority of instances, immediate death is caused 1*st*, by *Carbonic Acid Poisoning*; 2*d*, by *Carbonic Oxide Poisoning*; 3*d*, by *Carbon Suffocation.*

In some cases these influences are combined.

Carbonic Acid Poisoning is distinguished on *post-mortem* examination by the dark, semi-coagulated condition of the blood, by pulmonary congestion, and by the heart cavities being surcharged, (especially the right), with dark colored and soft blood coagula.

Carbonic Oxide Poisoning is distinguished mainly by the condition of the blood in the heart and large vessels, and by the absence of marked pulmonary congestion. The cardiac blood presents the appearance of masses of brick-red, waxy like matter, which does not alter on exposure to the atmosphere, while the pulmonary vessels, and even the capillaries, contain a similar material.

This condition of things, when it first came under my observation, somewhat puzzled me, but, on mentioning the matter to my colleague, Dr. Aitken, he informed me that the phenomenon was well known to chemists, and that it is due to the formation (by the action of the carbonic oxide upon the blood coloring matter) of insoluble compounds, which are not influenced or re-dissolved by exposure to the oxygen of the air.

This change in the blood most certainly exercises an important influence upon the system in some of those cases in which animals rally from the primary effects, but succumb to the secondary effects of exposure to an atmosphere charged with carbonic oxide; the normal lesion being of such a grave character as to be incompatable with the continuance of life.

Carbon Suffocation is due to the minute bronchii and air cells becoming plugged with finely divided particles of carbon in the form of smoke.

The accumulated carbon prevents the interchange of oxygen and carbonic acid, and the animal dies of Oxygen Starvation.

In *post-mortem* examination the particles of carbon can be distinguished in the form of black lines arranged in a precise? manner in the small tubes, the blood and heart presenting the same appearance as in the case of carbonic acid poisoning.

In Death Produced by Actual Burning the cauterized skin has a shriveled, charred appearance, while the fasciculi of the exposed muscles, in the horse at least, are rendered very distinct, almost as much so as if the inter-fascicular tissue had been carefully removed by dissection. The carcass presents much the same appearance as that already described as being characteristic of death by lightning.

*The Secondary and Remote results of exposure to Fire, Hot Air, Steam and Smoke* are more numerous, and on the whole, of more importance than are the primary.

They are, inability to rally from the effects of the primary shock, ante-mortem cardiac clotting, odema, (especially caused by hot air and steam) of the laryngeal tracheal and bronchial mucous membrane, sloughing of the mucous membrane, acute pulmonary congestion, and, in some cases, pulmonary hæmorrhage, bronchitis or broncho-pneumonia, emphysema of the lungs, cardiac derangement, nasal gleet, ophthalmia, enteritis, hœmaturia, tetanus and dermatitis with cellulitis.

Inability to Rally from the Primary Effects of Shock. In some cases, especially in horses, the vital forces though apparently annihilated for a time, are sufficiently powerful to enable an animal to throw off the immediate effects of the shock, but the depression of nerve force is so great as to render the carrying on of the functions of life an impossibility.

I am convinced, however, that in many instances this inability to rally is not due to nerve depression only, but, and probably in a much larger degree, to the grave physical and chemical blood changes which result from the inhalation of carbonic maroxide and dioxide.

In all cases of this kind, prompt and energetic treatment is demanded. Every effort should be made to rouse the vital energies, and to prevent cardiac asthenia.

A plentiful supply of fresh air should be allowed, general and special nerve stimulants administered internally, (brandy and ammonia *par excellence*), and active stimulating agents, as mustard or ammonia liniment, freely and repeatedly applied to the chest and legs. Stimulating inhalations and enemas are also useful.

Ante-Mortem Cardiac Clotting may take place within a few hours after exposure, or not until after the lapse of several days or even a week or two. It is due, in part to the weakened cardiac action, partly to the altered (spastic) condition of the blood, and in some cases, is favored by pulmonary congestion. The indications of the occurence of this process are, great irregularity and fluttering of the heart's action, rapid, weak and very irregular pulse, marked pre-pectoral bruit, and on auscultation, a blowing sound is heard over the region of the valves. There is great uneasiness, and probably profuse perspiration, difficult and hurried breathing, and distension of the superficial veins.

Here again, prompt treatment, such as that above recommended, must be adopted. Digitalis, and, after the lapse of a short time, potassic iodide, are especially useful in dealing with this condition.

ODEMA OF THE MUCOUS MEMBRANE OF THE AIR PASSAGES results more largely from steam, than from dry, hot air. It takes place with great rapidity, and if very extensive, produces death within a few hours by asphyxia.

It may or may not be complicated with *Effusion of Serum,* if it is, the gravity of the case is enormously increased. *Nasal, laryngeal and tracheal stertor* becomes pronounced, and it is quickly followed by paroxysmal coughing, and the discharge of frothy serum from the nostrils; this, in some cases, is mixed with blood, and if smoke has been inhaled along with the steam, the odor of charred matter is distinctly detected in the expired air.

Recovery, from such a condition of matters, is scarce to be expected; nevertheless, hope need not be abandoned, even though the animal may be pulseless, and all chances of recovery apparently gone.

Carbonate of ammonia can be given repeatedly in the form of a ball, (drenching is out of the question), stimulating enemas thrown into the rectum, and stimulating liniments applied to the thorax, and over the region of the trachea.

Tracheotomy, low down, may be performed with the object of faciliting the outflow of the serum.

If the animal rallies *sloughing of the mucous membrane* is still to be dreaded, and if the œdema has involved large tracts of bronchial membrane, death by asphyxia inevitably ensues.

ACUTE PULMONARY CONGESTION may occur immediately, or within a comparatively short space of time after the exposure, or not until the lapse of two or three days. It is a common experience that horses sent out to perform their ordinary work after being allowed to rest for a day or two, subsequently to the exposure, become the subject of this lesion; the symptoms becoming very pronounced; and if proper treatment is not promptly carried out, they quickly succumb to its effects. In some cases the engorgement is so great as to lead to *throwing out of blood* into the bronchial tubes, and its discharge through the nostrils, either in a fluid or in a semi-coagulated state, and very dark in color. Here again the odor of charred organic matter in the breath is very pronounced.

The adoption of measures equally as prompt as those recommended for cardiac clotting are demanded in the latter case, in spite of the best efforts, and though life may be prolonged for several days, or even for a week, gangrene is almost certain to result.

Bronchitis and Broncho Pneumonia are the natural sequelæ in those cases where the primary influences have not been sufficiently powerful to produce œdema or pulmonary congestion.

Bronchitis may or may not be preceded by or associated with *nasal catarrh.* It does not, as a rule, make its appearance before the expiration of thirty-six or forty-eight hours, and is most likely to result from exposure to a cold or cold and damp atmosphere.

Its symptoms and results do not differ in any material degree, except in the odor of the breath, from those of bronchitis, due to ordinary causes, neither is there anything special to be noted in reference to treatment.

*The most important results of Bronchitis* are broncho-pneumonia, chronic interstitial bronchitis and bronchonhœa.

Broncho Pneumonia, runs the same course and terminates in the same manner, though more likely to result in gangrene, as when arising from other causes.

If evidence, such as fœtid breath, of gangrene is forthcoming, the prognosis should be very unfavorable, as it is almost certain to terminate in death, but, notwithstanding this, antiseptic and stimulating treatment should be persevered with. Inhalations of terebene and creasote will asepticize the gangrenous products; stimulants and nutrients will support the vital powers until the *debris* has been got rid of.

Chronic Interstitial Bronchitis leads to thickening of the mucous and sub-mucous tissue, and by diminishing the calibre of the bronchioles, produces *asthma,* (one form of it), or as it is generally designated, *thick wind.* If this condition is not properly treated, it may render an animal useless for life, a least in so far as its power of performing hard work is concerned.

It may be cured or at least relieved, by the judicious administration of arsenic and potassic iodide, with nux vomica and careful feeding.

Bronchorhœa results from chronic catarrhal inflammation of the mucous membrane. Its duration is indefinite, and during its continuance an animal is practically useless. I have been very successful in arresting it by the application of a vesicant, such as Cautheim's ointment over the thorax and front of the chest; the administration of iodine, alternated with arsenic, and the inhalation twice daily of iodine fumes.

Pulmonary Emphysema may be *primary or secondary*; it is most likely to be the former, in cattle, owing to the histological difference which exist in the air cells and connecting tissue of the lungs, as compared with other animals, and in them is produced very rapidly and pronouncedly, in all conditions where there is material interference with respiration; e. g. in cattle plagues, exposure to foul air in the holds of ships, or to the effects of fog.

Secondary Emphysema may be compensatory in its origin, and due to organic destruction of parts of the lungs.

Cardiac Derangement is one of the most important and most frequent results of exposure to the influence of fire. It is most common in the horse and may result primarily from fright, secondarily, from important valvular or muscular changes. When resulting from fright it is due to the impression made upon the cardiac nerves themselves, or probably to muscular strain, produced by the violent action of the heart. *Palpitation* being present in a marked degree, in all animals rescued from burning buildings. The violent rebound of the heart may be sufficient, in some cases, to produce laceration of one or other of the valves, or of the chordæ tendinæ, and this supposition is warranted by the fact that these lesions are occasionally met with in horses and dogs that have been subjected to the effects of great and sudden excitement, violent strains or paroxysmal coughing; and by the further fact that one of the most common and important results of the action of fire and smoke in *valvular disease* either in the form of *tricuspid incom-*

*petence*, producing *jugular pulse or mitral obstruction*, producing *intermittence.* I am quite aware that the former may result from *dilatation of the auricular ventricular orifice* brought about by *dilitation of the ventricle*, and that the latter may be caused by the formation of *clots*, or even *vegetations* on the *bicuspid valve.*

I have sometimes thought that the *cardiac weakness* occasionally detected some weeks after the date of the exposure might be accounted for on the supposition that *fatty metamorphosis* of the *muscular fibrillae* had taken place in consequence of the organic nerve derangement.

While in some cases the judicious use of digitalis, nux vomica and ammonia, is successful in removing some of the symptoms above mentioned, it cannot be impressed too much upon the minds of practitioners that *cardiac derangement is one of the most important, most frequent, and most enduring of the sequelæ of exposure to the effects of fire and smoke.*

Nasal Gleet is a natural result of the primary nasal catarrh so often produced by hot air, steam and smoke. If it is properly treated in the early stages, it does not, as a rule persist; should it do so, it can, in the great majority of cases, be arrested by iodine fumigation and by the administration of such astringents and stimulants as iron and copper sulphate, and cantharides.

Trephining may be necessary, and should be had recourse to if milder measures fail in effecting a cure.

Superficial Ophthalmia is the direct result of the action of fire itself—of hot air, or of the gaseous and solid products of combustion. If the cornea is primarily damaged symptoms of *keratitis* are immediately observed, and, in addition, there is ocular evidence of the loss of corneal structure; otherwise, *conjunctivitis* may not be established until the lapse of several hours, or even a day or two, after the exposure. If the inflammation has been caused by actual flame, confirmatory evidence is to be found in singed eyelashes and scorched lids—the latter, too, are much tumefied, sometimes everted, sometimes closed, this depending upon the presence or absence of Odema, and its extent when present.

The catarrhal inflamation of the conjunctiva may, if not promptly treated, continue for many weeks; as a rule it disappears in a few days, especially if venesection (of the angular vein)

is resorted to with the application every few hours of a solution of cocaine—1 grain to the drachm,—and, after the lapse of two or three days, the alternate application of a collyrium of acetate of zinc. Should corneal ulceration result, the cocaine—as an anæsthetic—should be continued, and the ulcerated structure protected from the atmosphere by the occasional use of oleum amygdylæ dulc., medicated with 2½ p. c. of salicylic acid. Nitrate of silver or sulphate of zinc may be had recourse to if their use is medicated.

INTERNAL OPHTHALMIA is seldom seen, except the cornea is actually destroyed; in the dog, however, the great nervous excitement resulting from exposure to fire may originate *acute glaucoma.*

AMAUROSIS is an occasional result of extreme nervous excitement—it does not always yield to nerve stringli.

ENTERITIS is occasionally seen in the horse, not only as a sequel of exposure to actual fire, but as the result of the too severe application of the actual cautery in the operation of firing. In the most severe cases it is of the *phlegminous* type; but where it is due to continued irritation of the skin it is usually localized in the mucous membrane alone.

HŒMATURIA is less frequently seen than is hœmatinuria, and, when it does exist, is probably the indirect result of the latter condition, and is brought about by the irritant effects of abnormal matter from the blood passing through the kidneys and setting up violent congestion and *renal hemorrhage.*

Hœmatinuria is probably the result of an altered condition of the hœmato-globulin, brought about by the action of carbonic oxide and other deleterious, gaseous products of combustion.

Both of the above mentioned phenomena are of grave significance, pointing, as they do, to profound alteration of the physical and vital properties of the blood (especially the red corpuscles and their contents) in the one case, and to profound organic derangement of the kidneys in the other.

TETANUS is a remote result of cauterization, and is seen not only after exposure to fire, but, as in the case of enteritis, from the too severe use of the firing iron.

In the practice of Mr. R. Rutherford three or four cases occurred in one stud of horses.

Dermatitis associated or not, according to the severity of the cauterization, with *cellulitis*, is an inevitable result of exposure to the actual effects of fire, as also of hot water, and even of super-heated steam. In some cases, the inflammation is superficial, and confined to the extremities, and most largely in horses secured in stalls, to the hind limbs. If, however, the flame has run along the racks and mangers, the skin of the head, breast, and fore arms receives the greatest amount of damage.

Pathologically, neither dermatitis nor cutaneo-cellulitis differ materially from that produced by other causes, such as the too severe use of counter-irritants or exposure to caustic acids or caustic lime, and such combustible material as pitch, petroleum, etc. Cases of the latter kind, it may be remarked in passing, being sometimes experienced in horses engaged in chemical works and new erections.

In the worst forms of injury extensive *sloughing* takes place, the resulting wounds being of such large area as to preclude the possibility of healing under a shorter period than several months. I have seen the healing process extend to eight and ten months; this is, especially the case where the loss of structure is localized in the angles of flexion or in parts subjected to stretching, as between the thighs.

In the practice of Messrs. Lawson, of Manchester, I saw, only last year, a cart horse suffering from acid cauterization of the skin of the internal surface of the thighs, and, in that case, the healing process was indefinitely retarded by the horse's own movements, in spite of such mechanical restraints as sling, stocks, and fetters.

Deformity, produced by the contraction of the cicatrix in the healing process, is another result to be dreaded, and, if possible, guarded against in the healing process of extensive wounds. It must be prevented, when practicable, by the use of mechanical leverage secured by splints, plasters, bandages, lever-toed shoes, etc., as occasion demands.

Even in those instances where actual destruction does not take place, the skin becomes so enormously swollen as to cause great suffering by extreme tension, and to produce deep cracks or fissures and extensive suppuration with, in the end, the formation of ugly and disfiguring cicatrices.

The lips are sometimes so much swollen and cracked as to interfere with prehension; the nostrils are sometimes closed by the resulting tumefaction, rendering, in the horse, the operation of tracheotomy necessary. The labia of the vulva, in the female, and the anus become so much swollen in some cases as to produce marked eversion of their respective mucous membranes.

Death may be caused: 1st. From the *great nervous excitement*, followed by *exhaustion*, produced by the pain of the cauterization; and 2d. By exhaustion from *fever and subsequent suppuration.*

The cardinal principles of treatment of Burns and Scalds are: 1st. To protect the exposed surfaces from the irritant effects of the atmosphere and the germs it contains; 2d. To relieve the pain and irritation as quickly as possible; 3d. To support the system, and thus prevent exhaustion; 4th. To circumscribe sloughing and moderate suppuration; 5th. To favor the process of cicatrization, and, in doing so, to prevent deformity.

The first indication is carried out by the immediate application of a layer of cotton-wool, flour, starch, arrow-root or other material, which will either protect the part absolutely from the air, or filter it, and thus remove irritating particles.

From a painful experience, I am enabled to assert positively that nothing gives so much relief as the immersion of the injured part in any bland fixed oil; unfortunately this cannot well be carried out in veterinary practice.

For large, superficial burns, the best application is the well-known "Carron Oil," to which may be added carbolic acid (1 in 12), or prussic acid (1 drachm to every 4 ounces). Liquor plumbi may be used with advantage as a substitute for lime water; and, contrary, though it may seem, turpentine liniments give great relief.

*The second indication* is practically met by the adoption of the measures recommended in the first.

In addition, however, to local application, systemic measures for the relief of pain should be promptly adopted. Large doses of morphia should be injected sub-cutaneously, and continued until the irritation is relieved. If pain and irritation continue exhaustion inevitably results.

*The third indication* is carried out by the judicious administration of easily digested nutriments and stimulants—brandy or port wine in preference.

*The fourth indication* can only be effectually fulfilled by the free use of antiseptics; pads of tow, oakum, or cotton-wool saturated with creasote liniment or glycerine. Carbolic acid should be kept in close apposition, by means of bandages or other appliances, with the exposed parts.

A paste of starch, glycerine and carbolic (or salicylic) acid is also very useful for this purpose.

Lastly, the process of cicatrization may be favored by the formation of an artificial scab. Probably there is no combination which fulfils the various indications as to treatment of burns and scalds so perfectly as a mixture of equal parts of liquor plumbi, comp'd tincture of catechu and terrebene.

Where there is much cracking of the superficial portions of the skin a little glycerine may be added to the mixture. I have sometimes seen the best results from the free application of znic ointment, but no matter what measures are adopted, the process of cicatrization in extensive injuries must necessarily be a tedious one. In some cases the healing process is facilitated by the existence of little patches (islets) of undestroyed skin left over the surface of the wound—each one of these acting as a formative centre, from which cicatrization goes on in a radiating direction.

In concluding my remarks on this subject, it may not be considered out of place if I give a few hints as to the rescue of animals from burning buildings, and as to the duty of veterinary surgeons when called upon to give reports as to the damage done to animals by fire, smoke or steam.

*Rescue of Animals.*—The greatest difficulty in effecting a rescue is always experienced in dealing with horses. The terror induced by fire or smoke in these animals renders them, in some cases, *frantic* and *uncontrollable*; in others, *paralyzed* and *bewildered.*

There can be no more miserable or exciting experience than that of witnessing the state of terror into which horses are thrown under these circumstances, and it is intensified when no means can be devised for effecting a rescue.

The wild neighing, the snorting, stamping, kicking and plunging of the victims, followed by stifled groans and moans as death approaches, make up a horrid and horrifying experience that few humane persons care to pass through more than once in their lives. The difficulty of rescue is sometimes aggravated by a curious tendency on the part of some horses to free themselves from their rescuers, and rush back into the burning stables. Such incidents are not uncommon, and serve to show the necessity of removing horses to a safe distance from the scene of a conflagration with all possible speed.

Various methods of dealing with refractory horses have been practised. Some animals will, with a little coaxing, walk out of a stable at once; others require to be blindfolded; others, again, can be coerced by the free use of the whip. Some can be backed out, while harness horses will, in the majority of instances, walk quietly out after the harness is put on.

In dealing with obstreperous horses, all available forms of force should be brought into requisition, and if ropes are at hand (with plenty of manual assistance) they should be placed round the chest and hind quarters, and the animals dragged out by main force. In the case of cattle, the necessary amount of traction may be applied by a rope placed round the horns.

In those instances where the flames have not reached the imprisoned animals and where, from surrounding circumstances, it is impossible to effect a rescue, provision should be at once made for the conveyance to them of a full supply of fresh air by breaking through windows, doors, or even walls. I have seen a number of good horses lost by the neglect of this simple precaution. I am quite aware that in some cases the establishment of a current of air will tend to aggravate the evil by increasing the activity, and favoring the spread of the flames, but there are numerous instances in which much good can be effected in this direction.

The Duty of the Veterinary Surgeon in acting as a skilled arbiter between an insurance company and an insurer is sometimes both an unthankful and an invidious one. He is, however, only called upon to use ordinary care in estimating the immediate and remote effects of such accidents; and in accordance with his knowledge of their probable effects upon the systems of the injured animals; to estimate to the best of his ability not only the existing, but also by the concurrent and the sequential lesions—what will be the ultimate extent of the damage sustained. In some instances this estimate can, from the nature of the cases, be only a speculative and an approximate one. I am of opinion that the best and most satisfactory method of procedure—so far as living animals are concerned—is to submit those that are injured to a careful examination by two veterinary surgeons—one representing the owner, the other the insurance company, and let them advise as to the desirability of immediate slaughter, or of the adoption of curative measures in each individual case. A second examination should be held after a sufficient time has elapsed to enable them to judge as to the advisability or otherwise, of continuing the application of remedial measures. In all severe cases of burns and scalds, and of lung injuries, immediate slaughter is both the most humane and most profitable course to pursue.

